# Constitutive expression of the sRNA GadY decreases acetate production and improves *E. coli* growth

**DOI:** 10.1186/s12934-015-0334-1

**Published:** 2015-09-18

**Authors:** Alejandro Negrete, Joseph Shiloach

**Affiliations:** Biotechnology Core Laboratory, National Institute of Diabetes and Digestive and Kidney Diseases, National Institutes of Health, Building 14A Room 173, Bethesda, MD 20892 USA

**Keywords:** Acid resistance, sRNA, GadY, Glutamate decarboxylase, Acetate

## Abstract

**Background:**

*Escherichia coli* responds to acid stress by applying various physiological, metabolic, and proton-consuming mechanisms depending on the growth media composition, cell density, growth phase, pH, and aerobic or anaerobic growth conditions. It was reported that at mild acidic conditions (pH 5.8), the Hfq-associated sRNA GadY is activated. It was also reported that the two decarboxylase systems—the lysine decarboxylase system (LDS) and the glutamate decarboxylase system (GDS)—are activated to maintain intracellular balance of protons. The purpose of this study was to establish the role of GadY in high density growth of *E. coli* and to evaluate the possibility of using this small RNA to create an acid-resistant strain suitable for industrial applications.

**Results:**

Parental *E. coli* K-12 and constitutively expressing GadY strains were grown to high cell densities in a bioreactor at pH 7.0 and pH 6.0. At pH 7.0, both strains grew to similar cell densities of 43 OD, but the constitutively expressing GadY strain produced around 6 g/L acetate compared with 10 g/L by the parental strain. At pH 6.0, the parental strain grew to an OD of 20 and produced 10 g/L of acetate while the GadY strain grew to an average OD of 31 and produced 4 g/L acetate. After analyzing 17 genes associated with acid stress, it was found that at pH 7.0 LDS was expressed in the early exponential phase and GDS was expressed in the late exponential phase in both strains. However, at pH.6.0, GDS was expressed in the late exponential phase only in the parental strain and not in the constitutively expressing GadY strain, while there was no difference in the LDS expression pattern; it was expressed in the early exponential phase in both strains. This indicates that GadY affects GDS expression at low pH since the GDS was not detected in the GadY strain at pH 6.0.

**Conclusions:**

The constitutive expression of GadY improves *E. coli* growth at pH 6.0 by deactivating the expression of the GDS in the late exponential growth phase. The expression of GadY also decreases acetate production regardless of pH, which decreases the inhibitory effect of this acid on bacterial growth.

## Background

Growing *E. coli* to high cell densities is the preferred method for large scale production of recombinant proteins [1]. During this process, the bacteria are being exposed to various stress parameters that can possibly affect their growth and production capability. Examples of stress parameters are dissolved oxygen (DO) concentration, pH, temperature and osmolality, as well as substrates such as glucose, and metabolites like acetate and ammonia [2]. To prevent potential growth inhibition issues, there is a continuous effort to create bacterial strains that are resistant to different stress conditions and to develop growth strategies that will minimize stress [3].

It was established that exposing *E. coli* to acid stress triggers physiological and metabolic changes and activates proton consuming systems [4]; each consists of two cytoplasmic decarboxylases that catalyze a proton-dependent decarboxylation reaction of an amino acid, and a membrane antiporter that exchanges external substrate for internal product [5]. Two proton-consuming acid resistance systems active at mild acidic conditions (pH 4.0–5.7) have been described in *E. coli*: (1) the lysine decarboxylase system (LDS) that is activated in the early exponential growth phase and consists of the enzyme CadA and the lysine/cadaverine antiporter CadB [5–7], and (2) the glutamate decarboxylase system (GDS) that is induced in the late exponential phase and is comprised of the isozymes GadA, GadB, and the glutamate/γ-aminobutyric acid (GABA) antiporter GadC [8, 9]. Two additional proton consuming systems have been described: (1) the arginine decarboxylase system (ADS) that includes the AdiA enzyme and the arginine/agmatine antiporter AdiC which is activated in extreme acid environments under anaerobic conditions [5], and, (2) the ornitine decarboxylase system (ODS) which include the SpeF enzyme and the ornithine/putrescine antiporter PotE that is not well understood [5, 10].

Small RNAs are found to be involved in *E. coli* exposed to different environmental stresses such as temperature, pH, nutrients concentration and oxygen level [11, 12]. These are non-coding RNA molecules comprised of 50–250 nucleotides; and so far approximately 70 have been identified experimentally [13]. The most studied sRNAs are known to bind to the chaperone Hfq protein for pairing with the target mRNA [14]. The regulatory effects of the Hfq-associated sRNAs are translation repression (e.g. OxyS in oxidative stress), translation activation (e.g. DsrA in low temperature), mRNA degradation (e.g. SgrS in glucose internalization), and mRNA stability (e.g. GadY in acid stress) [11, 14, 15].

In this study, constitutive expression of the sRNA GadY was evaluated as a possible regulator of acid resistance in *E. coli* grown at high glucose concentration to high density. GadY was selected since the three forms of GadY (GadY 105, GadY 90, and GadY 59) were expressed when *E. coli* was grown at pH 6.0 in shake flasks [15]. GadY activates the acid resistance genes *gadA*, *gadB*, and *gadC* which are part of the GDS, and positively regulates the target GadX mRNA, which induces the expression of the GDS [15–17]. Depending on the media and the growth phase, GDS can also be induced by 11 regulatory proteins (RpoD, RpoS, EvgAS, YdeO, GadE, GadX, GadW, Crp, TrmE, HNS, and TorR) [5]. The growth characteristics, acetate production and gene expression profile of the strain constitutively expressing GadY (GadY strain) are described in this report. The results suggest that this modified strain can resist the stress caused by lower pH and high acetate concentration.

## Methods

### Strains

The parental strain is *E. coli* K-12 MG1655 (F-, λ-, ilvG-, rfb-50, rph-1); the strain that constitutively expressed sRNA GadY (pRI-GadY) is MG1655 that was modified by Dr. Gisela Storz from the National Institute of Child Health and Human Development [15]. The pRI plasmid genotype consists of a PKK177-3 expression vector with an EcoRI site at the transcription start site (Amp^r^). The complete description of the creation and characterization of the strain are available in the original reference [15].

### Bacterial growth

Bacteria were grown in modified LB media containing 10 g/L tryptone, 15 g/L yeast extract, 5 g/L NaCl and 5 g/L K_2_HPO_4_ at 37 °C. After sterilization, MgSO_4_ was added to a final concentration of 10 mM, 1 ml/L of trace elements was added and the glucose concentration was adjusted to 40 g/L [18]. A five liter bioreactor was inoculated with an overnight culture to an initial OD_600_ of 0.3, pH was controlled at 7.0 by 50 % (v/v) NH_4_OH, and the dissolved oxygen (DO) was controlled at 30 % air saturation. Bacterial density was measured at OD_600_ with Ultrospec 3000 UV/V spectrophotometer (GE Healthcare Bio-Sciences, Pittsburgh, PA, USA). Samples were collected and centrifuged at 13,000×*g* for 5 min and the cell pellet and the supernatant were kept at −80 °C for RNA extraction and metabolites analysis. For acid stress experiment, the culture was grown at pH 7.0 to the middle of the exponential log phase; at that point the pH was reduced to 5.0 with 0.5 M acetic acid for a period of 2 h and then was adjusted back to pH 7.0 with 50 % (v/v) NH_4_OH. For the growth experiments at pH 6.0, the culture grew at initial pH 7.0 without pH control until the pH decreased naturally to pH 6.0 and was maintained constant with 50 % (v/v) NH_4_OH. For evaluating the pH effect on bacterial growth, the pH of the culture grown at pH 7.0 was adjusted to 5.0 with 0.5 M acetic acid or with 0.5 M phosphoric acid at a cell density of 2 OD. Experiments were performed in duplicates.

### RNA extraction

The Hot phenol method was used for RNA extraction [18]. The cell pellets were resuspended in 0.5 % SDS, 20 mM sodium acetate and 10 mM EDTA and extracted twice with hot acid phenol: chloroform (5:1, pH 4.5) followed by two extractions with phenol:chloroform:isoamyl alcohol (25:24:1). Ethanol was added and the mixture was kept at −80 °C for 15 min before centrifugation at 14,000×*g* for 15 min; the pellets were washed with 70 % (v/v) ethanol, air dried and resuspended in ultrapure water. Total RNA concentration was estimated by measuring optical density at 260 nm using the NanoDrop 2000/2000c spectrophotometer, and integrity was visualized on a 2 % agarose gel.

### Northern blot analyses

Northern blot analyses were performed as described previously [18]. 5 μg of total RNA were separated on a TBE 10 % urea polyacrylamide gel (BioRad, Hercules, CA, USA) and transferred to a positively charged nylon membrane (BioRad, Hercules, CA, USA). A 5′-biotinylated sRNA-specific probe and the Bright-Star^®^ Biodetect™ nonisotopic kit (Life Technologies, Grand Island, NY, USA) were used for probing and detection. The membranes were probed, washed and conjugated with streptavidin–alkaline phosphatase using the BrightStar^®^ Biodetect™ Kit. Chemiluminescent signals were detected using the Fujifilm LAS-4000 imaging system. The 5′–3′ biotinylated sequences of the sRNAs probes used were reported previously [19].

### Determination of transcript levels by RT-qPCR

The total RNA extracted was treated with the Turbo DNA-free™ kit (Life Technologies, Grand Island, NY) to remove contaminating DNA. The RNA sample was incubated with the Turbo™ DNAse buffer and the Turbo™ DNAse at 37 °C for 30 min. The DNAse inactivation reagent was added to the RNA sample, incubated at room temperature for 5 min, centrifuged and the supernatant containing the RNA free of DNA was collected. The cDNA was synthesized using Maxima first strand cDNA synthesis kit for RT-qPCR (Cat. No. K1671, Life Technologies, Grand Island, NY, USA). The RNA was incubated with the Maxima enzyme mix and the template RNA at 25 °C for 10 min and then at 50 °C for 15 min. The reaction was stopped at 85 °C for 5 min. The genes analyzed were from the GDS (*gadA, gadB, gadC*), AGS (*adiA*), LDS *(cadA, cadB, cadC, IldC*), and other reported acid resistance genes (*gadX, gadE, slp, hdeA, hdeB, hdeD, ydeO, ydeP, and rpoS*). The *rrsA* of the 16S rRNA was selected as the normalizing gene. The primer pairs used for the RT-qPCR assay have been reported previously [20]. RT-qPCR was performed in 40 amplification cycles with each specific primer pair using SYBR^®^ Green PCR Master Mix (Life Technologies, Grand Island, NY, USA) as signal reporter. Reactions were run on an ABI Prism 7900H Sequence Detection System. Each reaction contained 600 ng cDNA and 400 nM of sense and antisense primers in a 20 μL reaction volume. The amplification parameters used for the RT-qPCR were 1 cycle at 95 °C for 10 min, 40 two-step cycles at 95 °C for 15 s and 1 cycle at 60 °C for 1 min. Each sample was analyzed in triplicate. No template and no reverse transcriptase controls were included. The expression of the *rrsA* gene was used as an endogenous control to normalize the amount of mRNA obtained from a target gene. Data was analyzed using the 2^−ΔΔCT^ method reported elsewhere [19]. The expression obtained for each time-point was normalized to the expression of each gene obtained in the parental strain under same conditions.

### Metabolite analysis

Glucose and glutamate were determined by the YSI 2700 SELECT Biochemistry Analyzer (YSI Life Sciences, Yellow Springs, OH, USA). Acetate was determined by HPLC, Hewlett Packard 1100 Series using an Aminex^®^ resin-based HPX-87H column (Bio-Rad, Hercules, CA, USA). The analysis conditions were as follows: the wavelength was 210 nm, the mobile phase was 0.008 N H_2_SO_4_, the flow rate 0.6 mL/min, and the temperature 35 °C, and utilizing organic acid analysis standard for calibration (Bio-Rad, Hercules, CA, USA). The non-dissociated form of acetate was calculated using the Henderson–Hasselbalch equation [21].

Cadaverine was determined by ion-exchange chromatography as described previously [22], using 4.6 mm internal diameter, 3.8 cm Shim-pack column No. ISC-05/S0504 (Shimadzu, Columbia, MD, USA) at 70 °C and a standard of 5 nM of cadaverine at a flow rate of 0.7 mL/min. The eluting buffer was 1 M NaCl, 0.2 M sodium citrate and the excitation and emission wavelengths were 360 and 430 nm, respectively. The post column fluorometric determination was done by reaction with fluoraldehyde OPA (*o*-phthalaldehyde) solution (Thermo Scientific, Rockford, IL, USA), collecting data using the PowerChrom 280 system (eDAQ Pty Ltd, Colorado Springs, CO, USA).

## Results

Parental and GadY *E. coli* strains were grown to high density in complex media in controlled bioreactor at pH 7.0 and 6.0. The growth parameters and the expression of genes related to acid stress response were measured and analyzed in early and late exponential growth phases. The genes evaluated by RT-qPCR were: *gadA, gadB, and gadC* from the GDS; *cadA, cadB, and cadC* genes from the LDS; and the *adiA* from the ADS. In addition, the following genes (not associated with a specific metabolic pathway) were also analyzed: *ldcC, gadX, hdeA, hdeB, hdeD, rpoS, slp, ydeO, ydeP, and gadE.*

1. GadY expression in the parental *E. coli* grown at pH 7.0 and exposed temporarily to pH 5.0

**Fig. 1:**
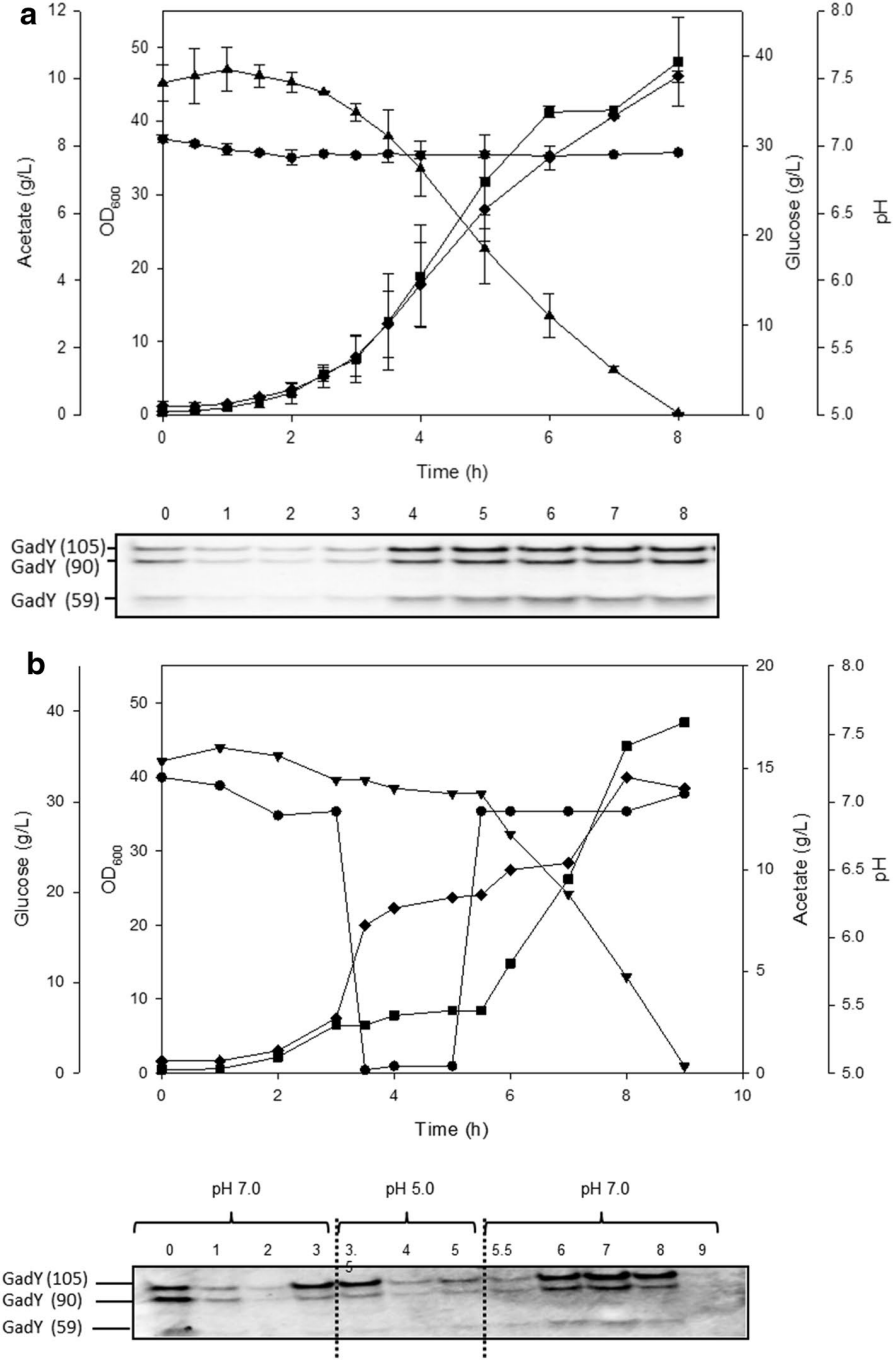
Cell growth parameters and GadY expression of *E. coli* MG1655 parental strain growing at **a** pH 7.0 and **b** pH 7.0 with temporal exposure to pH 5.0. *Filled square* OD_600_, *filled triangle* glucose (g/L), *filled diamond* acetate (g/L), and *filled circle* pH. The cells were grown in LB media in a 4L bioreactor. For the acid stress condition the pH was decreased to 5.0 by addition of 0.5 M acetic acid. After 2 h the acid stress was removed by increasing the pH back to 7.0. The expression of GadY was evaluated by Northern Blot loading same amount of total RNA for comparison purposes

**Fig. 2:**
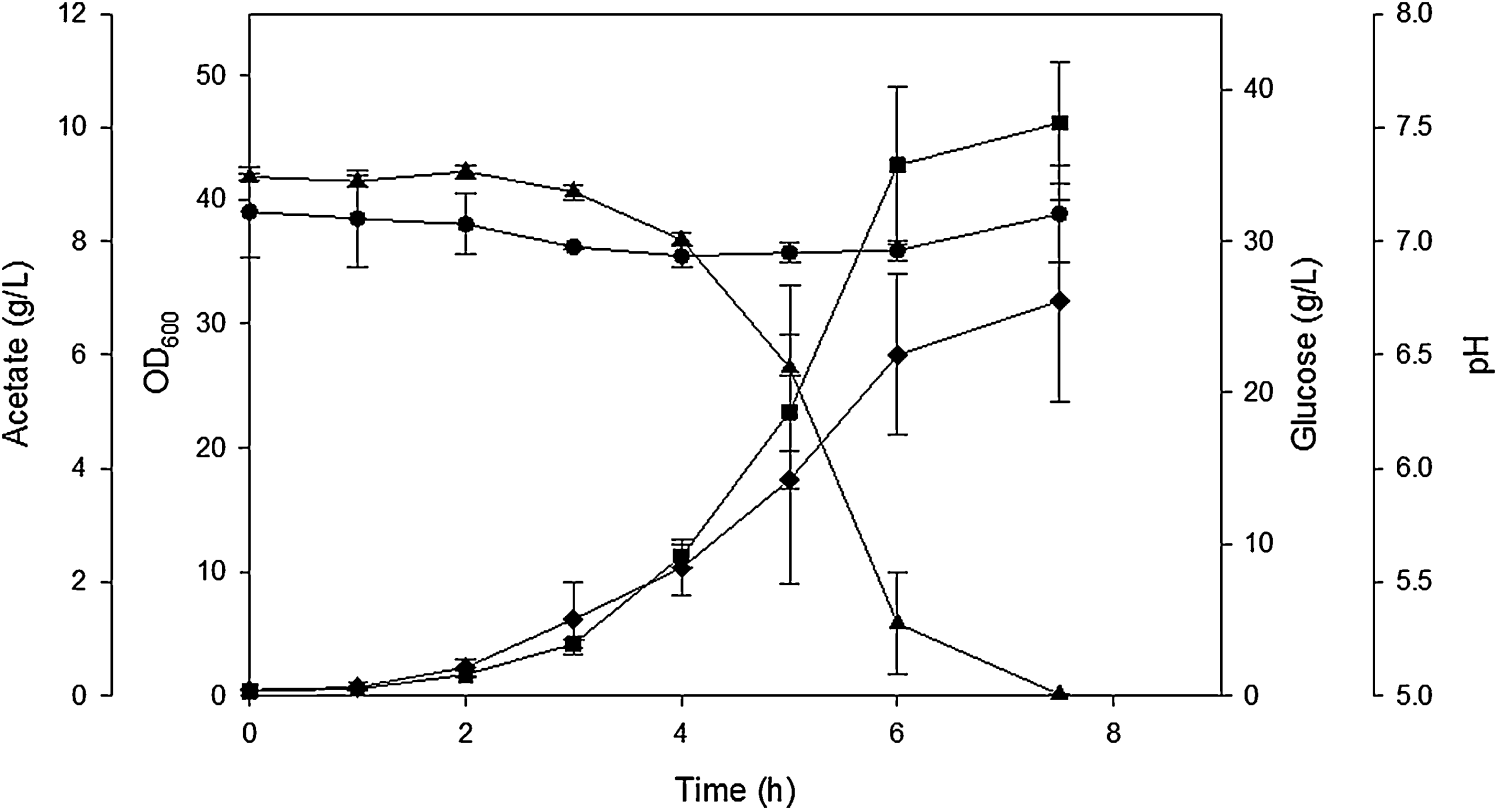
Cell growth parameters of *E. coli* MG1655 constitutively expressing GadY growing in LB media in a bioreactor at pH 7.0. *Filled square* OD_600_, *filled triangle* glucose (g/L), *filled diamond* acetate (g/L), and *filled circle* pH

To check the expression of GadY at high cell density culture, the parental strain was grown in a bioreactor at pH 7.0 and was exposed to pH 5.0 for 2 h in the middle of the exponential phase before adjusting the pH back to 7.0. The growth parameters and GadY expression are described in Fig. 1a, b. At pH 7.0, the three forms of GadY (GadY 105, GadY 90, and GadY 59) were expressed throughout the early and late exponential phases. This pattern was similar to the one reported when the bacteria was grown at pH 5.7 in shake flasks (Fig. 1a) [15]. However, when the pH of the growing culture was lowered to pH 5.0 for 2 h, the bacterial growth and the expression of GadY decreased considerably (Fig. 1b). The correlation between the GadY expression and the bacterial growth suggests that constitutively expressing GadY in *E. coli* may enhance the bacterial ability to overcome acid stress. To investigate this, GadY was constitutively expressed in *E. coli* and cultured at pH 7.0 (Fig. 2), the growth pattern was comparable to the parental strain (Fig. 1a); but acetate produced by the GadY strain was 6 g/L compared with 11 g/L produced by the parental strain.

2. Growth and acetate production patterns of parental and GadY strains at pH 6.0.

**Fig. 3:**
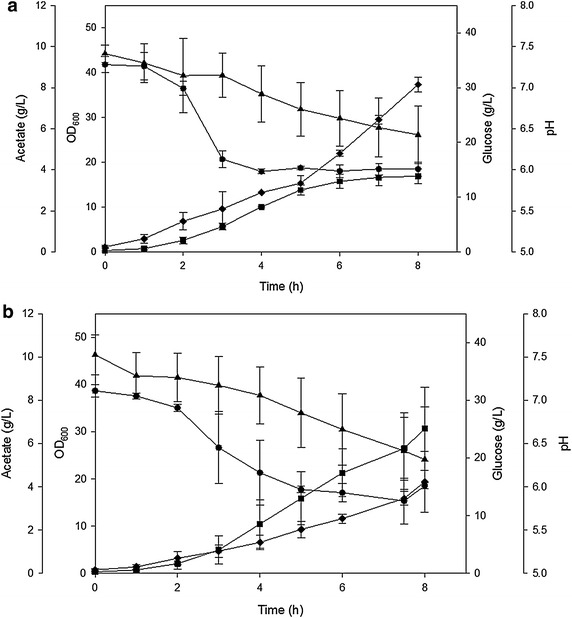
Cell growth parameters of *E. coli* MG1655 growing in bioreactor at pH 6.0 naturally decreased. **a** Parental strain and **b** GadY strain. *Filled square* OD_600_, *filled triangle* glucose (g/L), *filled diamond* acetate (g/L), and *filled circle* pH

Growth and acetate production patterns of the parental and the GadY strain at pH 6.0 are shown in Fig. 3a, b. At this condition, the GadY strain reached an average of 31 OD after 8 h of growth, while the parental strain reached 20 OD. At the same time, acetate production by the GadY strain was 4 g/L while the parental strain produced 10 g/L. This is an indication that constitutive expression of GadY improved the bacteria resistance to acid stress at pH 6.0; it reduced acetate accumulation and allowed the cells to grow to higher density.

3. Effect of acetic and phosphoric acids on the growth of the parental and GadY strains.

**Fig. 4:**
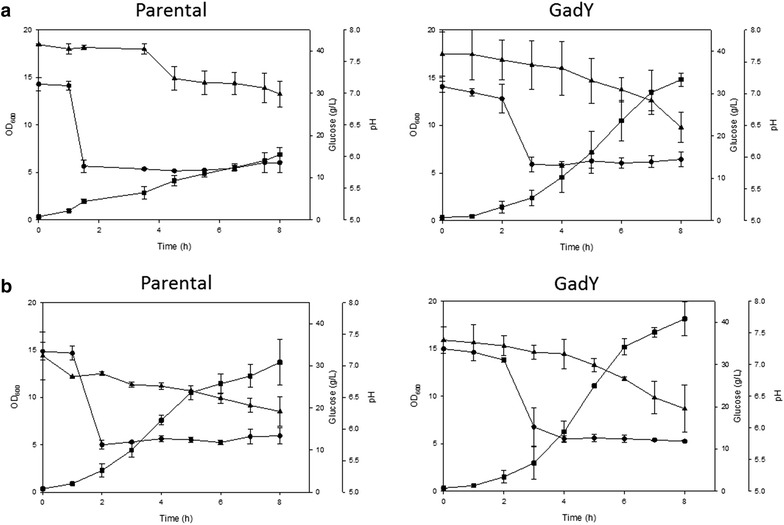
Cell growth parameters of *E. coli* MG1655 parental and GadY strains growing in bioreactor at pH 6.0. At OD 2.0 the pH was decreased from 7.0 to 6.0 by the addition of **a** acetic acid or **b** phosphoric acid. *Filled square* OD_600_, *filled triangle* glucose (g/L), and *filled circle* pH

The growth patterns of the parental and the GadY strains in media where the pH was adjusted to 6.0 by adding 0.5 M acetic acid, and in media where the pH was adjusted to 6.0 by adding 0.5 M phosphoric acid are shown in Fig. 4a, b. The results indicated that the parental stain was affected by the acetic acid but not by the phosphoric acid: it grew to an OD of 7 in the presence of acetic acid and to an OD of 14 in the presence of phosphoric acid. At the same conditions, the GadY strain grew similarly in presence of acetic acid and phosphoric acid; it grew to an OD of 15 in the presence of acetic acid and to an OD of 17 in the presence of phosphoric acid. The concentration of the non-dissociated acetate was determined and was found to be lower in the GadY strain than in the parental strain in both acid conditions (Table 1). The concentration of non-dissociated acetate was 57 and 88 mM for the GadY strain and the parental strain, respectively. This strain was not affected by acetate or by lowering the pH with phosphoric acid, an indication that GadY provides resistance to acetate and not to the effect of low pH.

**Table 1:** Total acetate and non-dissociated acetate concentration in late exponential phase of *E. coli* MG1655 parental and GadY strains grown at pH 6.0 by the addition of acetic acid or phosphoric acid

Strain	Acid added	Non-dissociated acetate (mM)	Total acetate (g/L)
Parental	Acetic acid	88	5.3
Parental	Phosphoric acid	68	5.4
GadY+	Acetic acid	57	5.3
GadY+	Phosphoric acid	39	4.4

4. Role of the GDS and LDS in the growth of parental and GadY strains at pH 6.0.

**Fig. 5:**
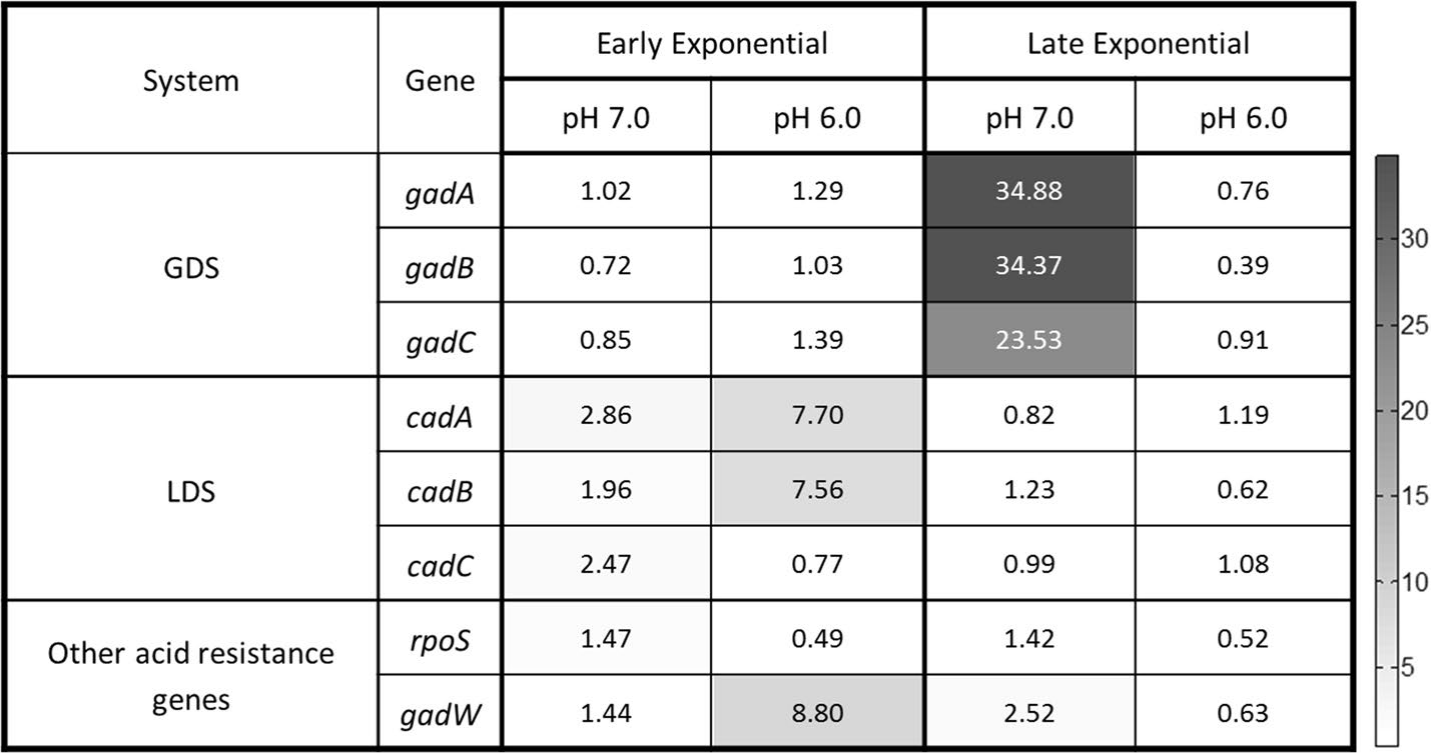
Expressed genes associated to acid resistance in *E. coli* MG1655 GadY strain. The gene expression was determined by RT-qPCR using the *ssrA* gene as internal control. The results are expressed in copy number using the parental strain grown under same conditions as reference

For the purpose of explaining the resistance to acetate and lower pH provided by GadY, the expression of the 17 genes reported to be associated with acid resistance was evaluated by RT-qPCR in the parental and in the GadY strains growing at pH 6.0 and 7.0. The expression ratio of the different genes between the GadY strain and the parental strain are presented in Fig. 5. The expression of LDS in early exponential phase and the GDS in the late exponential phase in the GadY strain grown to high density at pH 7.0 (Fig. 5) were found to be similar to the previously reported expression patterns of these two systems in *E. coli* grown at pH 6.0 [5, 7, 8]. However, when the GadY strain was grown at pH 6.0, the LDS was expressed in the early exponential phase as was observed in pH 7.0, but the GDS was not expressed in the late exponential phase, an indication that high cell growth of *E. coli* at low pH is independent of the expression of the GDS.

**Fig. 6:**
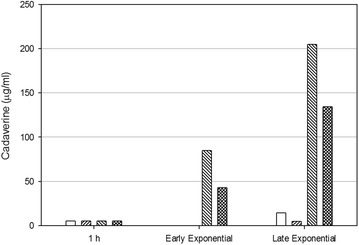
Cadaverine concentration determined in the media from early and late exponential phase of *E. coli* MG1655 parental and GadY strains. *White bars* for parental strain at pH 7.0, *right stripes* for GadY strain at pH 7.0, *left stripes* for parental strain at pH 6.0, and *checked bars* for GadY strain at pH 6.0

**Fig. 7:**
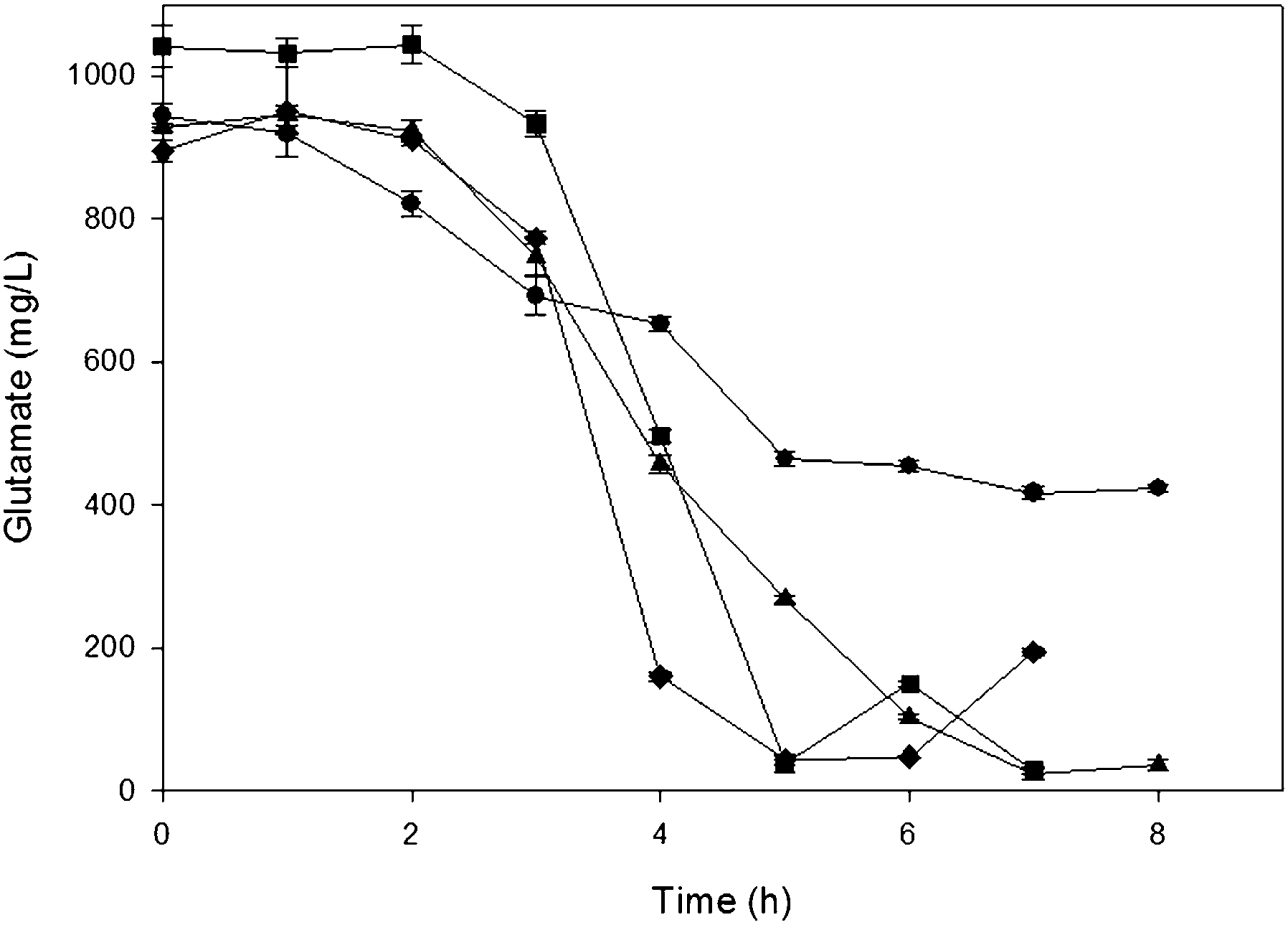
Glutamate consumption by *E. coli* MG1655 growing in bioreactor. *Filled square* parental strain at pH 7.0, *filled triangle* parental strain at pH 6.0, *filled diamond* GadY strain at pH 7.0, and *filled circle* GadY strain at pH 6.0

The results of gene expression of the LDS and the GDS were confirmed by measuring the cadaverine (Fig. 6) and glutamate (Fig. 7) concentrations in the growth media at early and late exponential phases of the two strains at both pH 6.0 and 7.0. The concentration of cadaverine in the early and late exponential phase was considerably higher in both strains at pH 6.0 than at pH 7.0 (Fig. 6), an indication that at pH 6.0 the LDS is more active than at pH 7.0. When the bacteria grew at pH 7.0, both strains completely consumed the glutamate in the media (Fig. 7). However, at pH 6.0, the partial consumption of glutamate by the parental strain growing at pH 6.0 was associated with the limited growth observed in Fig. 3a. The data obtained showed that the constitutive expression of GadY improves *E. coli* growth at pH 6.0 by activating the LDS and by decreasing acetate production, and consequently, minimizing the inhibitory effect of acetate accumulation.

## Discussion

The information presented in this study showed that compared with the parental *E. coli* MG1655 strain, the strain which constitutively expressed the small RNA GadY grew to higher OD at pH 6.0 and produced lower amounts of acetate at both pH 6.0 and 7.0. These results suggest that GadY has a role in the bacterial resistance to acid stress and in reducing acetate production. Acetate is well known to affect growth and recombinant protein production [23, 24], and, therefore, a strain constitutively expressing GadY may be beneficial for industrial application.

No significant difference was observed when the two strains were grown at pH 7.0 concerning bacterial density; however, acetate production from the GadY strain was about 40 % lower. When grown at pH 6.0, the GadY strain grew to higher than the parental strain and also produced less acetate. When evaluating the growth of the GadY strain and the parental strain at pH 6.0 that was generated either by acetic acid or by phosphoric acid, the GadY strain grew comparably in both acids while the parental strain was affected by the acetate and not by the phosphoric acid.

It was previously reported that growth inhibition and cellular damage are caused by the relative concentrations of dissociated acetate (CH_3_COO–) and non-dissociated (CH_3_COOH) acetate; where the non-dissociated form is more toxic [21, 23, 25–27]. It was determined that the concentration of the toxic non-dissociated acetate was higher (88 mM) in the culture of the parental strain which possibility caused the cells to grow to lower density in the presence of acetate than in the presence of phosphoric acid. On the other hand, the GadY strain reached similar growth in the presence of either acetic acid or phosphoric acid, likely as a result of the lower concentration of the toxic, non-dissociated from of acetate (57 mM).

The different response of the GadY strain to acid stress can be explained by expression of genes associated with acid stress, the *rpoS* gene, and the glutamate concentration. Response to acid stress in *E. coli* has been associated with expression of seventeen genes [20]. Therefore, the expression of these genes was measured in the parental and GadY strains grown at both pH 7.0 and 6.0. The analysis showed that GadY affected the expression of RpoS, GadW, and the genes of both the LDS and the GDS (Fig. 5).

**Fig. 8:**
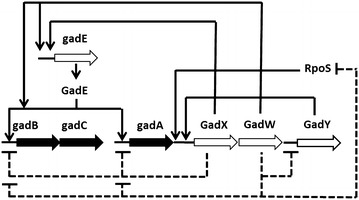
Regulatory network of the glutamate-dependent acid resistance. Modified from Foster, 2004. *Large arrows* represent genes, *small arrows* indicate positive control, and *dotted lines* denote negative control. The genes for the GDS are represented as *solid black arrows*

In the GadY strain grown at pH 7.0, the LDS was activated in the early exponential growth phase and the GDS in the late exponential phase, suggesting that both the LDS and the GDS have a role in maintaining intracellular homeostasis linked to acetate accumulation at pH 7.0. This differs from the induction of the GDS and the LDS described previously as acid stress response in *E. coli* grown at pH 5.8 [5, 7–9, 28, 29]. As observed in this work, the expression of the LDS and the GDS in the GadY strain grown at pH 7.0 is linked to acetate accumulation and not to low pH. The interaction between GadY and the GDS has been previously observed in an acid response regulatory network described in Fig. 8 [5, 15]. In that network, GadY induced GadX, which then activated GadW and the expression of the GDS directly or via *gadE*. In this study, GadY activated the GDS at pH 7.0 independent of *gadE*, *gadX*, and *gadW* as these three genes were not expressed. Also, it has been reported that the GDS can be induced either by acetate accumulation or by GadW via GadE in the presence of acetate [29–33]. Our results indicated that GadY activated GDS at pH 7.0 possibly, by an alternative mechanism to GadE, GadX, and GadW or by acetate accumulation.

Unlike the behavior at pH 7.0, GDS was not induced at pH 6.0 during the late exponential growth phase (Fig. 5), which disagrees with previous studies where GDS was activated in the late exponential phase regardless of the pH [5, 15]. This can be explained by the lower acetate concentration or by the effect of GadY on GadX and or GadW resulting with reducing GDS activation [16, 33–35].

The general stress regulator RpoS was not expressed in the GadY strain at pH 6.0 nor pH 7.0. The higher cell density reached by this strain compared to the parental strain at pH 6.0 suggests acid protective characteristics of GadY independent of RpoS. This agrees with previous reports where acid stress responses were induced in the absence of RpoS [8]. Another explanation for the non-expression of RpoS is that this global regulator may have degraded before it was analyzed [36].

Based on the presented observations, we hypothesize that the improved cell growth of the GadY strain is associated with glutamate concentration in the media. It is known that glutamate enters the TCA cycle providing 88 % of the cellular Nitrogen [37]. The higher cell growth of the GadY strain at pH 6.0 showed low concentration of glutamate, probably as a result of being metabolized by the TCA cycle to maintain cell growth. This hypothesis is supported by the growth profile of the parental strain at pH 6.0 which coincided with high concentration of glutamate in the media. The current study establishes a basis for the role of GadY in acid stress response at high cell growth and the benefit of incorporating this small RNA in *E. coli* to create a robust strain suitable for industrial application.

## Conclusions

Compared with its parental *E. coli* strain, the strain that constitutively expresses the small RNA GadY produced less acetate at both pH 6.0 and 7.0 and grew better at pH 6.0. Based on these growth properties, we consider this strain to be more suitable for high density growth in a bioreactor for industrial application. It was observed that at pH 7.0, the parental and the GadY strains grew similarly and the LDS was expressed in early exponential phase and GDS was expressed in late exponential phase. At pH 6.0, the GadY strain grew better and there was no expression of GDS at the late exponential phase. This strain produced less acetate at both pH levels. It was concluded that the protective effect of GadY is likely related to the concentration of the non-dissociated form of acetate and not to the low pH itself. These findings contribute to better understanding the role of sRNA GadY in acid resistance response at high cell density cultures.

## Abbreviations

LDS: lysine decarboxylase system
GDS: glutamate decarboxylase system
ADS: arginine decarboxylase system
ODS: ornitine decarboxylase system
GABA: γ-aminobutyric acid
DO: dissolved oxygen
OD_600_: Optical density at 600 nm
RT-Qpcr: quantitative real-time polymerase chain reaction
TCA: trichloroacetic acid
sRNA: small RNA
ssrA: small RNA encoding transcription unit used as internal control
